# Isolation and characterization of fetal nucleated red blood cells from maternal blood as a target for single cell sequencing‐based non‐invasive genetic testing

**DOI:** 10.1002/rmb2.12392

**Published:** 2021-06-14

**Authors:** Noriko Ito, Kazuhiro Tsukamoto, Kosuke Taniguchi, Ken Takahashi, Aikou Okamoto, Hiroaki Aoki, Yuka Otera‐Takahashi, Michihiro Kitagawa, Hiroko Ogata‐Kawata, Hideaki Morita, Kenichiro Hata, Kazuhiko Nakabayashi

**Affiliations:** ^1^ Department of Maternal‐Fetal Biology National Center for Child Health and Development Tokyo Japan; ^2^ Department of Pharmacotherapeutics, Course of Medical and Dental Sciences Nagasaki University Graduate School of Biomedical Sciences Nagasaki Japan; ^3^ Department of Obstetrics and Gynecology The Jikei University School of Medicine Tokyo Japan; ^4^ Aoki Obstetrics and Gynecology Clinic Tokyo Japan; ^5^ Sanno Birth Center Tokyo Japan; ^6^ Department of Allergy and Clinical Immunology National Center for Child Health and Development Tokyo Japan

**Keywords:** fetal nucleated red blood cell (fNRBC), fluorescence‐activated cell sorting (FACS), non‐invasive prenatal testing (NIPT), single cell, whole genome amplification (WGA)

## Abstract

**Purpose:**

Although non‐invasive prenatal testing (NIPT) based on cell‐free DNA (cfDNA) in maternal plasma has been prevailing worldwide, low levels of fetal DNA fraction may lead to false‐negative results. Since fetal cells in maternal blood provide a pure source of fetal genomic DNA, we aimed to establish a workflow to isolate and sequence fetal nucleated red blood cells (fNRBCs) individually as a target for NIPT.

**Methods:**

Using male‐bearing pregnancy cases, we isolated fNRBCs individually from maternal blood by FACS, and obtained their genomic sequence data through PCR screening with a Y‐chromosome marker and whole‐genome amplification (WGA)‐based whole‐genome sequencing.

**Results:**

The PCR and WGA efficiencies of fNRBC candidates were consistently lower than those of control cells. Sequencing data analyses revealed that although the majority of the fNRBC candidates were confirmed to be of fetal origin, many of the WGA‐based genomic libraries from fNRBCs were considered to have been amplified from a portion of genomic DNA.

**Conclusions:**

We established a workflow to isolate and sequence fNRBCs individually. However, our results demonstrated that, to make cell‐based NIPT targeting fNRBCs feasible, cell isolation procedures need to be further refined such that the nuclei of fNRBCs are kept intact.

## 1 INTRODUCTION

Fetal cells and fetal cell‐free DNA exist in the blood of pregnant women. ^1^, ^2^ The currently prevailing non‐invasive prenatal genetic testing (NIPT) is based on the massively parallel sequencing of cell‐free (cf) DNA from the plasma of pregnant women, and detects fetal chromosomal numerical abnormalities as well as small ( >5 Mb) sub‐chromosomal copy number changes. ^3^ The advantages of cfDNA as a target for NIPT are its abundance in maternal circulation and its ease of extraction from maternal plasma. However, as the low fetal fractions in cfDNA (5% to 20% depending on the gestational weeks) could lead to false‐negative diagnostic results, cfDNA‐based NIPT is regarded as a screening test that requires diagnostic confirmation by a conventional invasive test. ^3^ In addition, the short fragment sizes of cfDNA due to nuclease digestion limit the types of detectable fetal genetic alterations. Although they are very rare, circulating fetal cells in maternal blood, fetal nucleated red blood cells (fNRBCs) and trophoblasts, represent sources of fetal genomic DNA free from maternal DNA. ^4^ If these fetal cells are successfully isolated and their genomic DNA is amplified in a genome‐wide manner, definite detection of many types of genetic alterations including chromosomal inversions and translocations, and repeat expansions become possible in principle. Therefore, such a cell‐based NIPT is considered to have potential as a diagnostic test. ^1^, ^2^

fNRBCs were discovered in 1959, ^5^ and isolated for the first time in 1990. ^6^ The cells had been a primary target for NIPT research until cell‐free DNA was discovered. Fluorescence‐activated cell sorting (FACS) and magnetic‐activated cell sorting (MACS) were applied to separate fNRBCs from maternal cells and the separated cells were analyzed by fluorescent in situ hybridization (FISH) analyses. The National Institute of Child Health and Human Development Fetal Cell Isolation Study (NIFTY) was conducted to validate the utility of such FISH analyses for fNRBCs as a diagnostic method for fetal chromosomal abnormalities by recruiting 2744 maternal blood samples. ^7^ The detection ratios of fetal gender and fetal chromosomal aneuploidy were reported to be 41.4% and 74.4%, respectively, in 2002. ^7^ Because of the rarity of fNRBCs in maternal blood and the technical difficulty of consistently obtaining reliable results from the cells shown by the NIFTY study, the progress of cell‐based NIPT was stagnant until a few years ago.

Vossaert et al recently implemented a protocol for single circulating trophoblast testing using positive selection by MACS and single‐cell low‐coverage whole‐genome sequencing (WGS) to detect fetal aneuploidies and copy number variants (CNVs) at approximately 1 Mb resolution, and demonstrated the feasibility of utilizing their protocol as a form of NIPT. ^8^ Recent improvement in whole‐genome amplification (WGA) efficiencies ^9^ and single‐cell processing technologies, such as automated image capturing and single‐cell picking, ^10^ facilitated establishing a practical protocol for trophoblast cell isolation. He et al applied an immune‐affinity microchip ^11^ to isolate fNRBCs from maternal blood, and confirmed the fetal origin of the isolated cells by FISH analysis. Huang et al also conducted immune‐affinity capture of fNRBCs and trophoblasts based on their microfluidics technology, and subjected captured fNRBCs and trophoblasts separately as a pool to WGA. ^12^ They demonstrated that their WGA‐based array CGH and WGS both successfully detected fetal chromosomal aneuploidies. ^12^ However, such a pooled approach leaves the possibility of maternal cell contamination in fetal cells. On the other hand, single‐cell sequencing is more robust for such maternal contamination because sequencing data of fetal origin can be selectively used for genetic diagnostics after distinguishing the fetal or the maternal origin of each cell at data analysis procedures. Therefore, in this study, we isolated fNBRCs by single‐cell sorting and characterized the cells for their suitability for single‐cell genomics.

## 2 MATERIALS AND METHODS

### 2.1 Enrichment of NRBCs from maternal and cord blood and of lymphocytes from male blood

The blood samples were obtained using blood‐collection tubes containing EDTA‐2K. Blood from pregnant women (n = 9) or cord blood (n = 2) was diluted by adding two volumes of 1x phosphate‐buffered saline (PBS). Diluted blood was filtered through the Plasmodipur Filter (EuroProxima BV) to remove white blood cells. The filtered solution was layered on 1.5 volumes of Percoll solution with 1.119 g/ml density (Cytiva) in a 50 ml tube without disturbing the border of the two layers, and centrifuged at 400 × *g* for 30 minutes at room temperature (RT) to separate non‐nucleated RBCs and NRBCs by density gradient. ^13^ The resultant buffy coat layer containing NRBCs was transferred to a 5 ml tube, filled up to 4.5 ml with 1x PBS with 0.1% bovine serum albumin (BSA), filtered through a 35 μm cell strainer (#352235, Corning), and centrifuged at 400 × g for 5 minutes at RT. To completely remove non‐nucleated RBCs, the cell pellet was resuspended in 4 ml of BD Pharm Lyse lysing buffer (BD Biosciences) and incubated for 15 minutes at RT. After centrifugation at 400 × *g* for 5 minutes, NRBCs were further washed twice with 1000 μl of 1x PBS, and resuspended in 1000 μl of 1x PBS. To isolate control lymphocytes, male blood (n = 1) was processed in the same manner without using the Plasmodipur Filter.

### 2.2 Fluorescence‐activated cell sorting (FACS)

To stain dead cells, 1 μl of BD Horizon Fixable Viability Stain 450 (BD Biosciences) was added to the cell suspension and incubated for 12 minutes at RT. After centrifugation at 400 × *g* for 5 minutes at 4°C, the cell pellet containing NRBCs was resuspended with 500 or 1000 μl of 1x PBS with 0.5% BSA and 2 mM EDTA. After counting the cell number, the following amounts of antibodies were added to the cells: 4 μl of BD Pharmingen APC Mouse Anti‐Human CD71 (BD Biosciences), 0.8 μl of BD Pharmingen PE Mouse Anti‐Human CD235a (BD Biosciences), and 1 μl of FITC anti‐human CD45 Antibody (BioLegend) per 1 × 10^6^ cells. The cells were kept on ice for 20 minutes, centrifuged at 400 × g for 5 minutes at 4°C, and resuspended in 500 μl of 1x PBS with 0.5% BSA and 2 mM EDTA. After centrifugation at 400 × g for 5 minutes at 4°C, the cells were resuspended in 1500 μl of 1x PBS and filtered through a 35 μm cell strainer (#352235, Corning) prior to flow sorting. Cells showing the following fluorescent signal patterns, that is, CD71 positive, CD235a positive, CD45 negative, and Viability Stain 450 negative, were sorted using BD FACSAria III Cell Sorter (BD Biosciences). Lymphocytes from male blood were sorted by gating cells showing CD45 positive and other (CD71, CCD235a, Viability Stain 450) negative patterns. Individual cells were sorted into 96‐well plates (#0030129512, Eppendorf) preloaded with 18 μl of nuclease‐free water.

### 2.3 Real‐time PCR

Proteinase K (Merck KGaA) and 10x Single Cell Lysis & Fragmentation Buffer (Merck KGaA) were mixed at the ratios of 1:16. Two μl of the mixture was added to each well of the 96‐well plates containing a single‐sorted cell in 18 μl water. The plates were subsequently sealed with an adhesive film (#MSB1001, Bio‐Rad Laboratories, Inc). These procedures were conducted in a laminar flow cabinet. The sealed plates were subjected to cell lysis and fragmentation procedures (50°C for 60 minutes, 99°C for 4 minutes, and 4°C) on the Biometra TOne thermal cycler (Analytik Jena,). A portion of the cell lysate (1.8 to 3.0 μl) out of 20 μl was transferred to a new 96 well plate (#4346907, Thermo Fisher Scientific) and used as template DNA for the subsequent real‐time PCR using primers that amplify centromere repeat sequences. The original 96 well plates were stored at –20°C until use.

Two PCR primer pairs, one for DYZ3 centromeric repeats on Y chromosome (DYZ3F/R) ^14^ and the other for endogenous retroviral K111 repeats located at pericentromeric regions of 15 human chromosomes (K111F/R), ^14^ were used to detect only male cells and to detect male and female cells, respectively. For real‐time PCR amplification, 0.8 μl each of forward and reverse primers, 10 μl of Enzyme mix of TB Green Premix Ex Taq II (Tli RNaseH Plus) (Takara Bio), 0.4 μl of Rox Reference Dye II (Takara Bio) and 5 to 6.2 μl of Nuclease‐Free Water (17 to 18.2 μl in total) were added to the wells. The quantitative real‐time PCR data were obtained using Applied Biosystems 7500 Fast & 7500 Real‐Time PCR System (Thermo Fisher Scientific). The thermal cycling conditions used were 95°C for 10 minutes for template denaturation and initial activation of the enzyme, and 45 cycles of 95°C for 15 seconds and 60°C for 30 seconds, followed by a standard melting curve analysis.

### 2.4 Whole‐genome amplification (WGA) and NGS library preparation

Lysates of single cells that had already undergone cell lysis and fragmentation procedures were subjected to WGA and NGS library preparation using the SMARTer PicoPLEX Gold Single Cell DNA‐Seq Kit (Takara Bio) without its cell lysis procedure. When a portion of the cell lysate was used for PCR assays, the remaining cell lysate was used for WGA. Serially diluted cell suspensions (with cell densities of 1, 3, 10, and 30 cells per 5 μl) of a lymphoblastoid cell line (HEV0230) ^15^ and pooled NRBCs isolated from cord blood were also subjected to WGA and NGS library preparation.

### 2.5 Genomic DNA extraction and preparation of regular whole‐genome sequencing (WGS) libraries

Genomic DNA was extracted from 200 μl of maternal blood or umbilical cord blood using the QIAmp DNA Blood Mini Kit (Qiagen). Genomic DNA (600 ng) was sheared into fragments with an approximate average size of 350 bp using the DNA Shearing System S220 (Covaris), and subjected to NGS library preparation using the NEB Next Ultra II DNA Library Prep Kit for Illumina (New England Biolabs) with four PCR cycles.

### 2.6 NGS and sequencing data analysis

Regular and WGA‐based WGS libraries were sequenced using Illumina's sequencers, MiSeq, NextSeq550, or HiSeq X (Illumina) with paired‐end 150 bp and dual index settings. Read numbers varied depending on the purposes of the WGS analyses, that is, either to check the extent of amplification biases or to obtain a sufficient read depth for variant calling. Bam and gvcf files were created from fastq files using the DRAGEN (Dynamic Read Analysis for GENomics) Bio‐IT Platform v3.6.3 (Illumina). GRCh38 decoy was used as a human reference genome. Mapping and PCR duplicate rates and mean read depths were obtained from a report file generated by DRAGEN. The numbers of uniquely mapped reads for each of the chromosomes were counted from the bam file using the view command in samtools (http://www.htslib.org) with the option of “‐q 40” for filtering out reads with a mapping quality score (MAPQ) less than 40.

## 3 RESULTS

As positive controls for real‐time PCR screening, WGA, and WGS, we isolated 96 lymphocytes as CD45‐positive and CD71/CD235a/Viability Stain 450‐negative cells from an adult male blood sample in a 96‐well plate by single‐cell sorting (Figure S1). We assessed the efficiency of single‐cell PCR by DYZ3 and K111 primers with three different amounts of single‐cell lysate as template DNA, namely, 9.0% (1.8 μl) for 24 cells, 12.0% (2.4 μl) for 24 cells, and 15.0% (3.0 μl) for 24 cells. Among 72 cells (wells) tested, concordant results were obtained by two assays at 67 wells (93.1%), that is, not amplified by either of two primer pairs at five wells and amplified by both at 63 wells (Table S1A). The average Ct values (and the standard deviation, SD) obtained by K111 and DYZ3 primers were 35.8 (1.24) and 32.2 (1.63), respectively. These results demonstrated that the success rate of cell sorting was 87.5% (63 out of 72 wells tested). When we prepared WGA‐based single‐cell genomic DNA libraries using cell lysates of eight male lymphocytes, the yields of the final libraries ranged from 216.0 to 503.3 fmoles (Table S2). We also prepared WGA‐based WGS libraries using a female lymphoblastoid cell line and NRBCs isolated from cord blood as starting materials.

**FIGURE 1:**
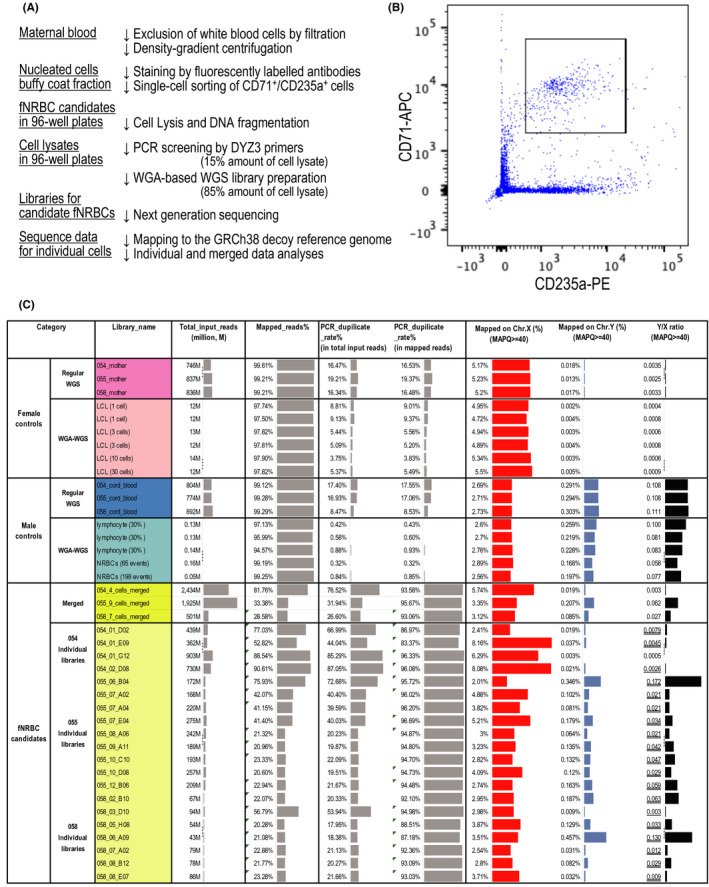
A. A workflow to isolate and to characterize single fNRBCs as adopted in this study. B. A FACS dot plot of nucleated cells isolated from blood of a pregnant woman (Case 058). Boxed CD71 and CD235a double‐positive cells were sorted. C. Mapping metrics of the sequencing data of the regular WGS and WGA‐based WGS libraries. Female control libraries include three regular WGS libraries prepared from 600 ng genomic DNA of maternal blood and six WGA–WGS libraries prepared from cells of a lymphoblastoid cell line (LCL). Male control libraries include three regular WGS libraries prepared from 600 ng genomic DNA of cord blood of the newborn, three WGA–WGS libraries prepared from 30% of the amount of the cell lysate of a single lymphocyte, and the WGA–WGS libraries prepared from pools of NRBCs isolated from cord blood (65 and 196 FACS events). Sequencing data of the WGA–WGS libraries prepared for fNRBC candidates were analyzed individually and all together for each case (“merged”). The Y/X ratio value of an fNRBC candidate was double undermined or single undermined, when it surpassed a threshold for the presence of Y chromosome, that is, the average Y/X ratio plus 3 SD value of the female control regular WGS libraries (0.0044), or that of the female control WGA–WGS libraries (0.0012)

We recruited nine pregnancy cases with a male fetus. Obstetrical complications or fetal abnormalities examined by fetal ultrasonography at 22‐37 weeks of gestation were absent in all cases. The ranges of gestational weeks and the amount of blood obtained 22‐37 weeks and 5.5‐12.0 ml, respectively (Table 1). For each maternal blood sample, we first removed white blood cells using the Plasmodipur Filter, subsequently immuno‐stained the remaining nucleated cells, and sorted CD71 and CD235a double‐positive cells as fNRBC candidates individually in the wells of 96‐well plates by FACS (Figure 1A, Figure 1B, Figure S1b). The number of double‐positive events ranged from 18 to 1121 (Table 1). We screened for male cells by PCR using DYZ3 primers, which amplify Y‐chromosome‐specific high‐copy tandem repeats. We used 15.0% amounts of single‐cell lysate for this PCR screening, and kept the rest for WGA. In total, we subjected single‐cell lysates of 3170 wells from nine cases to this screening, and identified 44 DYZ3‐positive wells (1.39% on average). The number and rate of DYZ3‐positive wells in each case ranged from 0 to 20, and 0% to 2.78%, respectively (Table 1). The average of the Ct values of 44 DYZ3‐positive wells was 38.1, which was 5.9 larger than that of male lymphocytes (32.2, Table S1A). However, peak patterns of the end‐point PCR products in the melting curve analyses were indistinguishable between those DYZ3‐positive wells and positive male DNA controls (6.6 pg DNA/well) (data not shown), ensuring the presence of male‐derived genomic DNA in those DYZ3‐positive wells. Therefore, we were able to identify fNRBC candidates by immuno‐staining and single‐cell sorting followed by PCR screening. However, single‐cell PCR efficiencies of fNRBC candidates were remarkably lower compared with those of control male lymphocytes.

**TABLE 1:** Recruited pregnancy cases with singleton male fetus and the summary of single‐cell isolation procedures

Case number	050	051	052	053	054	055	056	057	058
Gestational weeks (w) and days (d) at blood sampling	36w3d	35w6d	36w6d	36w0d	28w0day	28w4d	36w4d	22w5d	37w0d
Blood volume (ml )	7	7.5	9.5	7.5	7.5	12	5.5	5.5	9.5
Number of cells before cell sorting	3.9 × 10^5^	13.3 × 10^5^	9.7 × 10^5^	1.5 × 10^5^	11.9 × 10^5^	66.9 × 10^5^	9.3 × 10^5^	2.1 × 10^5^	32.1 × 10^5^
Number of CD71/CD235adouble‐positive cells	1104 (576)^a^	336	105	40	179	1121	18	36	759
Number of DYZ3 PCR‐positive cells (average Ct)	8 (38.9)	2 (37.3)	2 (37.8)	0 (NA)	4 (38.6)	20 (37.8)	0 (NA)	1 (38.2)	7 (37.8)
Ratio (%) of PCR‐positive cells per double‐positive cells	1.39%	0.60%	1.90%	0%	2.23%	1.78%	0%	2.78%	0.92%
Ratio of PCR‐positive cells per cells before sorting	20.6 × 10^‐6^	1.5 × 10^‐6^	2.1 × 10^‐6^	0	3.4 × 10^‐6^	3.0 × 10^‐6^	0	4.8 × 10^‐6^	2.2 × 10^‐6^
PCR‐positive cells per ml blood	1.1	0.3	0.2	0	0.5	1.7	0	0.2	0.7

^a^ 576 out of 1104 double‐positive cells were subjected to the DYZ3 PCR screening.

To further confirm the fetal origin of the fNRBC candidates and evaluate their quality as a source for WGA‐based WGS, we conducted a WGS analysis. WGA‐based single‐cell WGS libraries were prepared for 20 fNRBC candidates in total. Since 15.0% amounts of cell lysate were already used for PCR screening (Table S1B), the rest of the cell lysate was subjected to the WGA procedure. Consistent with the lower amplification efficiencies observed in the PCR screening procedure, the library yields of fNRBC candidates tended to be markedly lower (ranging from 2.0 to 87.2 fmoles) than those of male lymphocytes (Table S2). In total, we obtained 2434 million (M) reads from four cells, 1925 M read pairs from nine cells, and 501 M read pairs from seven cells for Cases 054, 055, and 058, respectively, by paired‐end sequencing (150 bp × 2) (the average read numbers per cell were 609 M, 214 M, and 72 M). We also prepared three each of regular WGS libraries for the mother's peripheral blood and the cord blood of the newborn of these three cases. Compared to the typical and consistent mapping rates (average 99.3% and 99.2%) and genome coverage (average × 31.3 and × 33.0) observed for the regular WGS libraries, those of single‐cell libraries were lower and variable in three cases: the mapping rates were 81.8%, 33.4%, and 28.6% and the genome coverage values were × 4.4, × 0.57, and × 0.18, respectively, for these cases (054, 055, and 058), when sequences from multiple single‐cells were combined in each case (Figure 1C, Table 2). These genome coverage levels obtained for fNRBC candidates were not sufficient to conduct further analyses, such as SNV and CNV detection.

**TABLE 2:** Mean and standard deviation (SD) of mapping metrics of control and fNRBC candidate libraries

WGS library type	Read numbers (million, M)	Mapping rate (%)	PCR duplicate rate (%)in total reads	Mean read depth	Mapped reads on X (%)MAPQ>40	Mapped reads on Y (%)MAPQ>40	Y/X ratioMAPQ>40
Female control libraries							
Regular WGS (n = 3)	806 M (43 M)	99.3% (0.2%)	17.3% (1.3%)	×31.3 (1.5)	5.20% (0.03%)	0.016% (0.002%)	0.0031 (0.0004)
WGA–WGS (1 to 30 cells; n = 6)	12.4 M (0.7 M)	97.8% (0.1%)	6.3% (2.0%)	ND	5.06% (0.27%)	0.004% (0.001%)	0.0007 (0.0002)
Male control libraries							
Regular WGS (n = 3)	823 M (50 M)	99.2% (0.1%)	14.3% (4.1%)	×33.0 (3.6)	2.71% (0.02%)	0.296% (0.005%)	0.109 (0.001)
WGA–WGS (1 cell & pooled cells; n = 5)	0.12 M (0.04 M)	97.2% (1.8%)	0.6% (0.2%)	ND	2.70% (0.12%)	0.214% (0.030%)	0.080 (0.013)
fNRBC candidate single‐cell libraries (WGA–WGS)							
WGA–WGS_054 (4 cell data merged)	2434 M	81.8%	76.5%	×4.4	5.74%	0.019%	0.003
WGA–WGS_055 (9 cell data merged)	1925 M	33.4%	31.9%	×0.57	3.35%	0.207%	0.062
WGA–WGS_058 (7 cell data merged)	501 M	28.6%	26.6%	×0.18	3.12%	0.085%	0.027

Note

SD is shown in parentheses.

**FIGURE 2:**
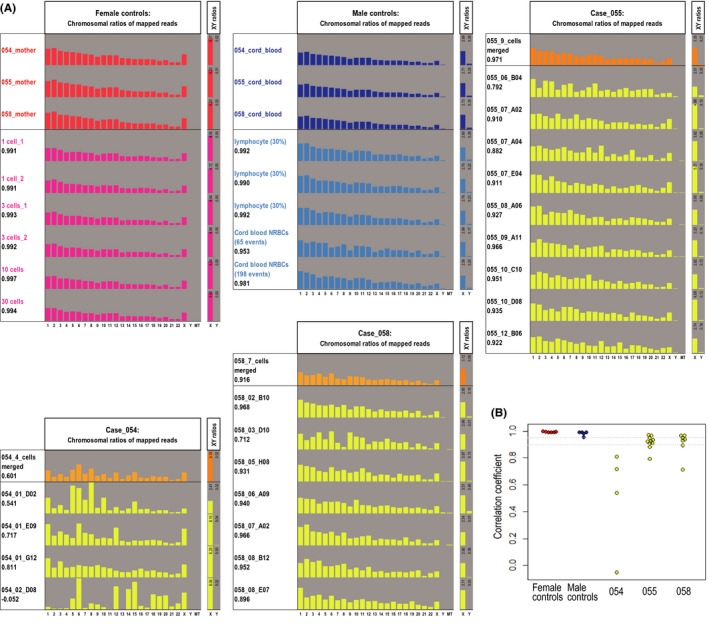
A. Chromosomal distribution of mapped reads observed in regular WGS libraries and WGA‐based WGS libraries. The chromosomal ratios of female and male control libraries are shown in red/magenta bars and blue/light‐blue bars, respectively. The chromosomal ratios of merged and individual data of fNRBC candidates are shown in orange and yellow bars, respectively. The vertical‐axis ranges for all chromosomes (1 to 22, XY, and MT) and for sex chromosomes are 0% to 20% and 0% to 6%, respectively. B. The correlation coefficient between the chromosomal ratios of mapped reads of a WGA‐based WGS library and the averaged ratios of control regular WGS libraries is shown for each WGA‐based WGS library. Female control WGA–WGS libraries were compared with the averaged ratio of three female control regular WGS libraries (“mother”). Male control and fNRBC WGA–WGS libraries were compared with the averaged ratio of three male control regular WGS libraries (“cord_blood”). Two horizontal dashed lines represent correlation coefficient levels of 0.90 and 0.95

To further confirm the male origin of fNRBC candidates using sequencing data, we examined the numbers of reads mapped to sex chromosomes. The average ratios of the reads mapped to Y chromosome to the reads mapped to X chromosome (Y/X ratios) were 0.0031 and 0.109 in female and male control regular WGS libraries, respectively (Table 2). The Y/X ratios were 0.0007 and 0.080 in six female and five male control WGA–WGS libraries, respectively (Table 2). The Y/X ratios of fNRBC candidate single‐cell libraries (WGA–WGS) were 0.003, 0.062, and 0.027 for Cases 054, 055, and 058, respectively, when sequencing data from multiple cells were merged (Figure 1C, Table 2). These intermediate values suggested the possibility that only a subset of fNRBC candidates were of male origin, or that the fNRBC candidate libraries was not amplified uniformly. When the average Y/X ratio plus 3 SD values of the female control regular WGS libraries and of the female control WGA–WGS libraries, which were 0.0044 and 0.0012, were used as thresholds to determine the presence or absence of Y chromosome in each individual single‐cell library, 17 and 19 out of 20 individual cells showed a higher Y/X ratio than the threshold, respectively (Figure 1C). To evaluate the uniformity of the WGA–WGS libraries of the fNRBC candidates, we calculated the correlation coefficient of chromosomal distribution of mapped reads between a WGA–WGS library and control regular WGS libraries. Whereas the WGA–WGS libraries of the female lymphoblastoid cell line consistently showed a correlation coefficient higher than 0.99, those of fNRBC candidates tended to be lower and more variable (Figure 2), that is, only 12 and 5 libraries showed a correlation coefficient higher than 0.90 and 0.95, respectively. These results indicate that the majority of the fNRBC candidate cells we isolated through our FACS‐based workflow were in fact of male fetal origin, but many of their WGA libraries showed poor library yields accompanied with non‐negligible amplification biases.

## 4 DISCUSSION

Isolation of single circulating fetal or trophoblast cells could revolutionize the field of prenatal diagnosis because these cells are pure sources of fetal DNA. Recently, Vossaert et al demonstrated that NIPT using trophoblast cells is feasible to detect copy number changes at 1 Mb resolution. ^8^ Considering confined placental mosaicism, fNRBCs are more suitable as a target for definitive NIPT than trophoblast cells. Previous studies,^11^, ^12^ that aimed to utilize fNRBCs in maternal blood for NIPT concentrated the cells, but did not completely remove maternal cells from fetal cells, leaving the possibility of inaccurate diagnosis due to maternal cell contamination. Therefore, we aimed to isolate single fNBRCs by cell sorting followed by real‐time PCR confirmation. Considering the wide prevalence of FACS in medical research institutions, an advantage of our workflow is that it does not require a special device dedicated to single‐cell isolation of fNRBCs.

The number of the cells we obtained by FACS as double‐positive cells for two erythroid‐associated markers (CD71 and CD235a) ranged from 18 to 1121 in nine cases tested (Table 1). The ratios of male fetal cells identified by DYZ3 PCR among double‐positive cells ranged from 0% to 2.78%. The frequencies of those cells per milliliter of blood ranged from 0 to 1.7 (median 0.3). This median value was consistent with the average number of male (XY) NRBC cells in maternal blood isolated by CD71 antibody selection and identified by FISH (3.38 +/− 1.7 cells from 10 ml of blood), recently reported by Nemescu et al.^16^

We adopted a strategy to screen for fNRBC candidates by real‐time PCR using a portion (15%) of single‐cell lysate before WGA. This is because cell‐based NIPT is still feasible even lacking a portion of genomic DNA of a cell by merging sequencing data from more than one cell. When 50% of the entire genome is covered without allelic dropout by the sequencing data from one cell on average, merged data from 5 and 10 cells could cover 96.9% and 99.0% of the genome, respectively. However, in both real‐time PCR and WGA‐based single‐cell WGS analyses, the DNA amplification efficiencies of fNRBC candidates were markedly lower than those of control male white blood cells, indicating the possibility that genomic DNA was damaged and degraded in fNRBC candidates.

NRBCs are commonly found in cord blood. ^17^ Hematopoietic stem cells (HSCs) differentiate into primitive erythroid cells (EryP). EryPs undergo cell divisions several times, and further differentiate to erythroblasts, which have lost cell division ability. Erythroblasts undergo enucleation, an asymmetric cell division involving extrusion of a pyknotic nucleus enveloped by the plasma membrane and generation of a reticulocyte. ^18^ Reticulocytes further become mature red blood cells. Erythroblast enucleation has been shown to require the establishment and maintenance of cell polarization mediated by PI3K. ^19^ It has been also shown that Rac GTPases and their effector protein mDia2 play significant roles in mouse fetal erythroblast enucleation by affecting the formation of the contractile actin ring in late‐stage erythroblasts. ^20^ It has been shown that 43% of fetal erythroblasts were being apoptotic, ^21^ and that a higher oxygen concentration in maternal circulation than in fetal circulation induces apoptosis of fetal erythroblasts. ^22^ Fetal erythroblasts may not survive or may be progressively damaged during cell isolation procedures. ^16^ To prevent loss of fNRBCs by apoptosis and enucleation, formaldehyde fixation of cells, which is shown to be compatible with WGA, ^9^ is expected to be effective. Streck tubes (Cell‐Free DNA BCT, Streck) contain a fixative that keeps cells intact for a few days. It has been demonstrated by Cayrefourcq et al that maternal blood sampling using Streck tubes did not interfere with the recovery of the circulating trophoblastic fetal cells in maternal blood through density gradient centrifugation or subsequent procedures such as WGA and exome sequencing.^23^ Therefore, replacement of EDTA tubes with Streck tubes for maternal blood sampling is expected to protect fNRBCs from their loss immediately after blood sampling and to improve DNA amplification efficiencies at latter procedures.

The addition of PI3K and Rac GTPase inhibitors to blood may also be effective through preventing the enucleation of fetal erythroblasts. ^19^, ^20^

As the purpose of this study was to demonstrate the feasibility of isolating single fNRBCs from maternal blood by FACS and to evaluate their suitability as a source of DNA for single‐cell genomics, we recruited only pregnancy cases with a male fetus and used a Y chromosome‐specific marker to screen for fNRBC candidates. Once we successfully establish experimental conditions to efficiently isolate intact fNRBCs and to obtain high‐quality single‐cell WGS data, our strategy will be also applicable to pregnancy cases with a female fetus by using K111 primers at the PCR screening stage and obtain WGS data for PCR‐positive single cells. In this setting, by obtaining maternal and paternal WGS data and using informative SNVs that distinguish maternal and fetal genotypes, it is possible to determine the origin of each of the PCR‐positive single cells even when both maternal and fetal cells coexist among PCR‐positive cells.

Maternal blood sampling for NIPT is typically performed in the first trimester (as early as 10 weeks) or in the second trimester (before 22 weeks). In our initial study design, we planned to compare sequencing data from fNRBC candidate cells and from cord blood cells to assess allelic dropout rates of WGA–WGS libraries of fNRBC candidates. Therefore, by taking into account an expected sampling timing of cord blood within a month or so, we choose 35 weeks or later as the timing of maternal blood sampling among six out of nine cases in this study (Table 1). He et al ^11^ isolated fNRBCs from 48 maternal blood samples, whose gestational week at blood sampling ranged from 10 weeks to 30 weeks, and observed the highest peak of the number of fNRBCs around 18 weeks. Nemescu et al ^16^ also isolated fNRBCs from 27 pregnancy cases, whose gestational week at blood sampling ranged from 12 weeks to 30 weeks, and observed the highest number of fNRBCs in a sample at 21 weeks of gestational age. These results in previous studies indicate the possibility that higher numbers of fNRBCs are isolated than those in the current study (Table 1) when we apply our workflow to the maternal blood samples at gestational weeks earlier than 22 weeks.

In this study, we demonstrated that fNRBCs in the maternal circulation can be selectively isolated by single‐cell sorting, and confirmed their fetal origin by real‐time PCR and WGS. However, the PCR and WGA efficiencies of single fNRBCs were remarkably lower compared with those of control single lymphocytes. Our results suggest that it is a critical step to develop single‐cell isolation conditions that keep fetal erythroblasts nucleated and non‐apoptotic to eventually establish our workflow as an fNRBC‐based NIPT.

## 5 HUMAN RIGHTS STATEMENT AND INFORMED CONSENT, AND ETHICAL APPROVAL

All the procedures were conducted in accordance with the ethical standards of the institutional ethical committee on human experimentation (institutional and national) and with the Helsinki Declaration of 1964 and its later amendments. This research was approved by the institutional review boards of the National Research Institute for Child Health and Development (IRB number 699) and Sanno Birth Center (16‐S‐22). Written informed consent was obtained from both the pregnant woman and her partner in all cases.

## CONFLICTS OF INTEREST

The authors declare that there are no conflicts of interest.

## Supporting information

Figure S1

Table S1‐2

## Data Availability

Sequencing coverage data in bigwig format are available for WGA‐based WGS libraries of fNRBCs and regular WGS libraries of control genomic DNA upon request.
