# Supplementary material for: Isolation and characterization of fetal nucleated red blood cells from maternal blood as a target for single cell sequencing‐based non‐invasive genetic testing
Source: Reprod Med Biol. 2021 Jun 14;20(3):352–60. doi: 10.1002/rmb2.12392 (PMC8254165; doi:10.1002/rmb2.12392)
Supplement: Supplementary file 1 — Figure S1 [file RMB2-20-352-s001.pdf]

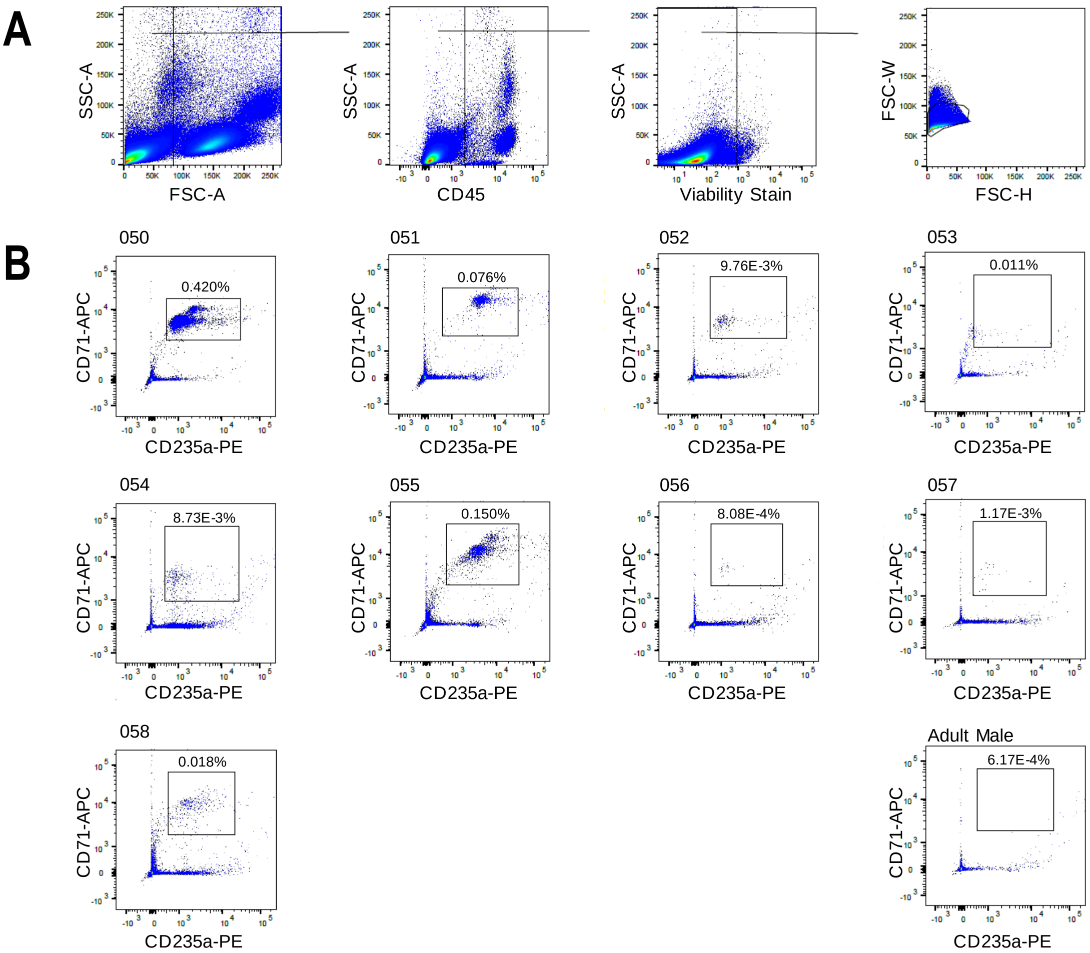

Figure S1: Gating strategy to sort CD71 and CD235a positive cells and FACS density plots for nine cases and a control.

A. Gating strategy to sort CD71 and CD235a double-positive cells..

B. FACS density plots of nucleated cells isolated from blood of nine pregnant women and of an adult male. CD71and CD235a double-positive cells are boxed.
